# Lignin Extraction by Using Two-Step Fractionation: A Review

**DOI:** 10.3390/molecules29010098

**Published:** 2023-12-22

**Authors:** Medya Hatun Tanis, Ola Wallberg, Mats Galbe, Basel Al-Rudainy

**Affiliations:** Department of Chemical Engineering, Lund University, P.O. Box 124, SE-221 00 Lund, Sweden; medya_hatun.tanis@chemeng.lth.se (M.H.T.); ola.wallberg@chemeng.lth.se (O.W.); mats.galbe@chemeng.lth.se (M.G.)

**Keywords:** two-step pretreatment, lignin extraction, fractionation

## Abstract

Lignocellulosic biomass represents the most abundant renewable carbon source on earth and is already used for energy and biofuel production. The pivotal step in the conversion process involving lignocellulosic biomass is pretreatment, which aims to disrupt the lignocellulose matrix. For effective pretreatment, a comprehensive understanding of the intricate structure of lignocellulose and its compositional properties during component disintegration and subsequent conversion is essential. The presence of lignin-carbohydrate complexes and covalent interactions between them within the lignocellulosic matrix confers a distinctively labile nature to hemicellulose. Meanwhile, the recalcitrant characteristics of lignin pose challenges in the fractionation process, particularly during delignification. Delignification is a critical step that directly impacts the purity of lignin and facilitates the breakdown of bonds involving lignin and lignin-carbohydrate complexes surrounding cellulose. This article discusses a two-step fractionation approach for efficient lignin extraction, providing viable paths for lignin-based valorization described in the literature. This approach allows for the creation of individual process streams for each component, tailored to extract their corresponding compounds.

## 1. Introduction

In recent decades, rising energy prices and the subsequent global energy crisis have underscored the necessity for immediate advancements in industrial applications. The ongoing disruptions in global supply chains, prompted by the pandemic, have further emphasized the need to adjust energy supply-demand strategies worldwide over the past few years [1]. In the context of environmental and energy security, reliability, affordability, and sustainability for industries, the biorefinery concept has gained prominence as a means to reduce global reliance on fossil-based industries and address climate challenges associated with them. It also plays a crucial role in supporting the implementation of related policies, such as the Glasgow Declaration on Sustainable Bioenergy [2]. A sustainable biorefinery is an integrated facility designed to produce a diverse array of biofuels, energy, bio-based chemicals, and value-added products, utilizing various conversion technologies, including thermochemical and biochemical processes, while utilizing lignocellulosic biomass as the feedstock [3]. Given its diversity, accessibility, carbon-neutral nature, and relatively low production cost, lignocellulosic biomass holds significant potential for various industries. Additionally, lignocellulosic biomass is vital to an integrated biorefinery due to its ability to generate value-added by-products and biofuels. The biomass-to-energy conversion process involves a diverse array of feedstocks, technology pathways, and resultant end products. In 2020, the World Bioenergy Association (WBA) reported that globally, electricity generation from biomass attained a level of 2.47 EJ. Notably, solid biomass sources and industrial and municipal wastes made significant contributions, accounting for 69% and 17%, respectively, of the total biomass-derived electricity generation. In addition to energy production, bioenergy has made substantial contributions to other sectors, including the transport industry, where biofuels accounted for 3.6% of overall energy consumption and renewable energy technologies represented 15% of the primary energy supply in 2020. Considering the global supply of biomass, which amounted to 57.5 EJ in 2020, solid biomass sources such as wood chips, pellets, and traditional biomass sources comprised 86% [4].

Lignocellulosic biomass, often comprising agricultural wastes and residues (e.g., straw, wheat, or bagasse), forest residues (wood sawdust, wood chips), lumber (softwood and hardwood), organic components of municipal waste, and energy plants, stands as the most abundant renewable carbon source on earth. At present, large quantities of lignocellulosic biomass are already employed in the pulp and paper industries [5], heat and electricity generation [6], and bioethanol production [7]. As mentioned earlier, lignocellulosic biomass exhibits significant potential for producing various value-added chemicals, fuels, fuel additives, and bioproducts [8,9] when suitable conversion technologies are applied. Moreover, its abundance, availability, and sustainability make it a vital component of the sustainable biorefinery approach. Since it is residual and nonedible feedstock, it is essential for ongoing discussions about the food and fuel dilemma [10]. Lignocellulosic biomass is composed of three main components: cellulose, hemicellulose, and lignin, in addition to various minor constituents, both extractives and non-extractives. It is important to note that lignocellulosic biomass is not homogeneous; its structures and chemical compositions vary based on the biomass’s source, species, or harvesting. Generally, it consists of 30–50% cellulose, 15–30% hemicellulose, 15–30% lignin, and 0–5% other minor components (such as proteins, esters/fatty acids, and inorganic materials) on a dry-weight basis [11,12]. Different types of lignocellulosic biomass exhibit varying proportions of these major constituents, as shown in Table 1.

*Cellulose*, a linear homopolymer composed of cellobiose linked together through β-1,4 glycosidic bonds, forms the crystalline fibrous structure and is relatively insoluble in most common solvents, including water, at low temperatures. Cellulose polymers are held together by intramolecular and intermolecular hydrogen bonds and Van der Waal’s forces generated by the accumulation of pyranose rings, thus forming crystalline microfibrils and acting as a structural scaffold [6,16]. *Hemicellulose*, a branched polymer containing pentoses (D-xylose and L-arabinose) and hexoses (D-mannose, D-glucose, and D-galactose), serves as an amorphous connector between cellulose and lignin. Hemicellulose can start hydrolyzing at lower temperatures compared to cellulose. Hemicelluloses consist of a limited number of sugar residues, typically shorter in length, with a degree of polymerization between 150 and 200. Both carbohydrates, hemicellulose and cellulose, are potential sources of fermentable sugars or other processes that convert sugars into products [6,14]. Lignin, often underestimated in the context of lignocellulose, is frequently considered a low-value by-product, and thermochemical conversion is the primary method of disposal due to its complex nature and its high calorific value. Lignin is a hydrophobic, well-branched, amorphous, aromatic natural polymer that provides structural integrity and protects against microbial degradation. Structurally, it is more complex compared to other components of lignocellulose [17].

Until recent years, the predominant approach to utilizing lignocellulosic biomass revolved around producing and commercializing biofuels and biobased chemicals from the pulp, with the primary focus on evaluating cellulose for further use [18,19]. To enhance saccharification yields and fermentability, reducing lignin content and collecting lignin at the end of the process were often preferred [20]. The complex structure of lignocellulosic biomass necessitates a pretreatment step to mitigate its recalcitrance and facilitate further conversion [21,22]. However, to completely utilize lignocellulosic biomass, it is imperative to evaluate all components of lignocellulose and develop viable methods for their valorization. Numerous studies examining different lignin valorization strategies and investigating various pretreatment techniques have highlighted lignin’s potential to address numerous challenges associated with fossil-based industries [23]. This review primarily focuses on lignin extraction; therefore, a detailed overview of lignin and its properties will be presented in the following.

**Figure 1:**
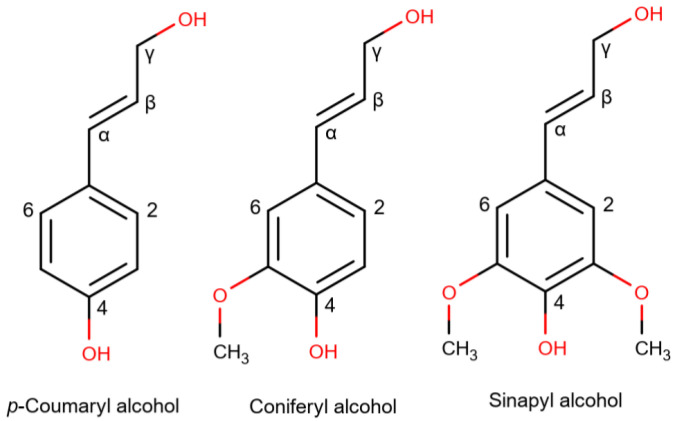
Monolignols of lignin [25].

Lignin stands as the second-most abundant carbon source on earth and holds significant potential as a natural source for various chemicals and valuable products. Lignin constitutes a mass fraction of 25–30% in softwoods, 19–28% in hardwoods, and 11–27% in nonwoody sources [24]. Lignin has a phenolic structure and consists of phenylpropanoids attached to this structure via side chains. It is a three-dimensional, heterogeneous biopolymer comprised of different phenylpropanoid units, specifically three types of hydroxy cinnamyl alcohol sub-units: coniferyl, sinapyl, and p-coumaryl alcohols, resulting in guaiacyl (G), syringyl (S), and p-hydroxyphenyl (H) units, respectively. These monolignols differ in the number of methoxy groups on the phenolic core, which can be seen in Figure 1, leading to variations in lignin’s structure and substructures, depending on the source. These units are interconnected via trifunctional linkages, creating branching regions that give rise to the characteristic network structure of lignin. Additionally, these structural monomers within lignin are interconnected by inter-unit linkages, primarily carbon-oxygen (C-O) bonds, with ether bonds (β-O-4, 4-O-5, and α-O-4) accounting for two-thirds or more. At the same time, the remainder consists of C-C bonds [12,16].

Softwood lignin predominantly comprises G units (approximately 90–95%) with a smaller fraction of H units (5–10%), while hardwood lignin mainly contains 25–50% G and 50–75% S units, with a smaller fraction of H units. Grass lignin consists of nearly equal amounts of G and S units, with notably fewer H units. Grass lignins exhibit a higher H unit content than softwood and hardwood lignins, as shown in Table 2. Guaiacyl lignin is known to be more chemically resistant than syringyl lignin due to the greater number of C-C bonds between guaiacyl units and other lignin units [26]. The S/G ratio in the cell wall has been shown to influence lignin degradability, with higher S-lignin content associated with increased lignin removal and higher sugar yields. The S/G ratio’s impact on lignin deconstruction and subsequent sugar release can be feedstock-dependent, as a lower metric has also been linked to higher sugar production. Plants with lower lignin content tend to facilitate enzymatic degradation by allowing enzymes greater access to cellulose [27].

The phenolic hydroxyl, benzylic hydroxyl, and carbonyl groups attached to the basic phenylpropanoid skeleton are considered the most influential functional groups in terms of the reactivity of lignin, allowing it to interact with a range of polymers and produce more homogeneous composite products. Moreover, β-aryl ether bonds are closely associated with lignin valorization, as breaking these bonds during lignin depolymerization leads to the formation of reactive intermediates, which subsequently create stable C-C bonds instead of hydroxyl groups, resulting in reduced reactivity of lignin sub-units compared to natural lignin. The abundance of phenolic hydroxyl groups in lignins contributes to their chemical modification and physical interactions [14,31,32]. Although methoxyl groups are relatively unreactive, they serve as an approximate measure of the phenylpropanoid content in a given lignin. The carboxyl and carbonyl groups of lignin have a strong tendency to form covalent bonds with specific chemicals, posing challenges in lignin applications and necessitating specialized technologies. Additionally, high polarity of lignin can lead to intermolecular interactions within composite structures. The potential for lignin-based co-products also depends on the inherent structures and chemical reactivity of lignin polymers [33,34,35].

Lignin properties can vary based on the source and extraction method. The predominant compositions in lignin, such as carbon and aromatics, render it more valuable, allowing for the development of value-added chemicals and bio-based materials. In recent years, as our understanding of lignin’s physical and chemical properties has improved, an increasing number of value-added products have been developed from lignin sources. In addition to its traditional use in the pulp and paper industry, research on lignin valorization has indicated the broad potential to create various end-uses, including bio-based materials [36], fuels and fuel additives [37,38], and value-added biochemicals [39]. The urgency to address current fossil-based industry challenges has also amplified the significance of lignin valorization. Nevertheless, the complex structure and heterogeneity of lignin present substantial barriers to its depolymerization and conversion into high-value-added chemicals. Multiple methods for further lignin fractionation have been explored, including alkaline [40], organosolv [41], ionic liquids [42], and deep eutectic solvents [43], to obtain specific molecular lignin fractions with defined properties. These efforts open new avenues for designing functional materials and provide promising opportunities for biorefineries. Efficient fractionation strategies, from lignocellulose to lignin, are crucial for the subsequent transformation into value-added products in the biorefinery platform. Many studies have examined selective fractionation technologies that add significant value to lignin and polysaccharides, recognizing their potential for comprehensive strategies. This approach underscores the need for fractionation technologies that can individually produce cellulose, hemicellulose, and lignin streams, offering a broad range of fuels, chemicals, and biomaterials [44,45]. However, one of the main challenges in selectively separating lignocellulose components is the potential conversion of carbohydrates, lignin, or both into unwanted byproducts during processing [31]. Therefore, more efficient methodologies for fractionating and preserving cellulose, hemicellulose, and lignin have been explored. The ‘lignin-first’ concept has emerged as an alternative approach to lignin valorization.

The ‘lignin-first’ biorefinery has garnered significant research interest as it enables the separation of lignocellulosic materials, producing high-quality pulps with low molecular weight lignin streams possessing controlled molecular diversity. It is important to note that this approach does not focus solely on lignin valorization; rather, it aims to obtain valuable products from both lignin and polysaccharides, promoting the efficient and sustainable utilization of lignocellulosic biomass. Lignin-first strategies typically involve the catalytic conversion of lignin during biomass fractionation, extracting lignin from biomass using solvolysis or acid catalysis extraction, followed by stabilization to prevent the condensation of reactive products resulting from lignin depolymerization. Subsequently, reductive stabilization of reactive intermediates is primarily carried out. The goal is to obtain high-purity lignin with favorable chemical reactivity. It is essential to address the degradation of hemicellulose, which often occurs during biorefinery processes and requires additional separation of the products from hemicellulose-derived C5 sugars and lignin-derived phenols, and consider its potential dissolution in lignin oil in the final phase [46,47,48,49]. Efforts to develop lignin valorization have shown that common single-step delignification pretreatments, such as ethanol organosolv, alkaline, and ionic liquids, can effectively extract lignin. Nevertheless, in many cases, the recovery of the hemicellulose fraction has been neglected, rendering it an impractical approach for evaluating all components of lignocellulosic biomass. Furthermore, the acidic conditions that solubilize hemicellulose can lead to the condensation of lignin, reducing its extractability and valorization potential. Given that lignin fractionation is especially important for value-added industrial applications and energy purposes, there is a growing emphasis on developing more efficient processes, such as a two-step pretreatment approach, to enhance lignin valorization. The complex nature of the lignocellulose matrix and the recalcitrance of lignin further justify the use of a two-step pretreatment approach, where hemicellulose is initially solubilized and recovered under mild conditions before lignin extraction. Most of the cellulose fraction remains in the cellulose-rich solid fraction, ready for further utilization, such as fermentation or hydrolysis [50,51].

The lignocellulose matrix comprises three primary types of bonds among its significant components: ether bonds, ester bonds, and carbon-to-carbon (C-C) bonds. The positioning and bonding of these linkages can vary, creating connections within the individual constituents of lignocellulose and interconnecting the different elements to form a complex structure [16]. Lignin, which encompasses various linkages with cellulose and carbohydrates, forms associations primarily with hemicellulose and disperses within the interstitial spaces of the matrix, creating a three-dimensional structure that enhances structural integrity. In contrast, lignin and hemicellulose are cross-linked through hydrogen and covalent bonds. These intermolecular lignin-carbohydrate (LC) chemical bonds between lignin and hemicellulose biopolymers are believed to occur naturally. As a result, cellulose, hemicellulose, and lignin intertwine to create a complex cell wall structure that resists biodegradation. Furthermore, the lignin-carbohydrate complex (LCC) poses challenges to the isolation of individual components, complicating the recovery process. Understanding the types and nature of these lignin-hemicellulose bonds is essential for the improvement of lignocellulose biorefinery [16,52].

Pretreatment plays a pivotal role in the conversion process, using lignocellulosic biomass to generate energy and biofuels, thereby facilitating the fractionation of lignocellulose. The micro- and macro-structure, as well as the chemical composition of lignocellulose, undergo changes during pretreatment. Historically, the primary objective of pretreatment was the release of polysaccharides from the lignocellulosic matrix to promote enzymatic cellulose hydrolysis, with scant attention given to potential hemicellulose and lignin utilization. The presence of LCCs, along with hydrophobic interactions and covalent bonds, results in the labile nature of hemicellulose and reinforces the recalcitrant nature of lignin. This complexity makes it challenging to fractionate these components using a single-step process. In the pretreatment phase, it is critical to avoid the degradation of major lignocellulose components for efficient fractionation. As previously discussed, the primary goal of pretreatment is to disrupt the lignocellulose matrix, facilitating the breakdown of lignin and LCC bonds surrounding cellulose. Consequently, lignin can be extracted and removed, depending on the specific objectives of the process. Simultaneously, hemicellulose degrades, enhancing cellulose availability and increasing enzymatic hydrolysis [53,54].

This review focuses on a two-step fractionation process for efficient lignin extraction and its utilization in lignin-based valorization pathways found in the literature. Furthermore, it investigates the impact of pretreatment methods, offering a comprehensive overview of the subject and insights into the potential products of the various components involved in the process.

## 2. A Short Summary of General Pretreatment Methods

**Figure 2:**
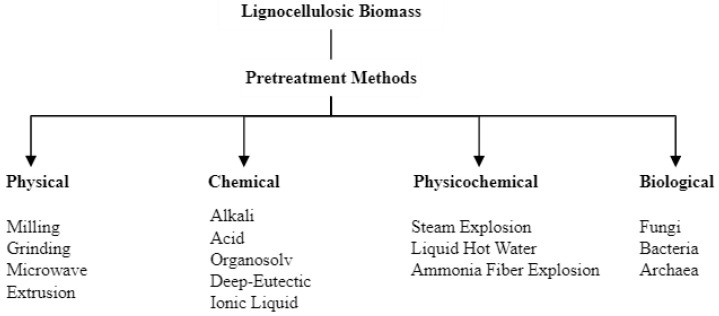
Various Pretreatment Methods for Lignocellulosic Biomass (Adapted from [54]).

As depicted in Figure 2, pretreatment methods are commonly categorized into physical, physicochemical, chemical, and biological. Traditionally, pretreatment of lignocellulose aimed to eliminate the hemicellulosic barrier surrounding cellulose and disrupt the lignocellulosic structure to enhance cellulose accessibility and enzymatic hydrolysis, primarily for bioethanol production. However, in recent years, the necessity of sustainable development and the need to reduce reliance on fossil-based industries for chemicals, fuel, fuel additives, or energy have underscored the importance of exploiting the individual components of lignocellulose for diverse product streams. When pretreatment targets diverse objectives, such as the production of bio-based polymers, fuel, or fuel additives, numerous pretreatment methods have been investigated. For instance, alkaline pretreatments enhance hemicellulose and cellulose accessibility, acidic conditions promote hydrolysis into simple sugars, steam pretreatment increases the accessible surface area by disrupting fibril structure, liquid hot water pretreatment facilitates disintegration and separation of lignocellulose, and organosolv processes promote lignin removal [55,56].

The key to effectively utilizing lignocellulosic compounds lies in selecting the optimal pretreatment method, taking into consideration the specific requirements of individual compounds and their corresponding final products. Additionally, an essential consideration during pretreatment is the intricate structure of lignocellulose and its compositional properties, as these components are broken down and converted. Furthermore, lignocellulose source and composition are not the sole determining factors in the pretreatment and subsequent biorefinery processes. There are various other parameters to consider, including energy consumption, cost, solvent requirements, the expense of catalysts, and operational costs, all of which are crucial for defining these processes in an industrial context [55,57,58,59]. Table 3 presents an overview of the advantages and drawbacks of these pretreatment methods.

### 2.1. Physical Pretreatments

Physical pretreatments are primarily employed for size reduction, enhancing storage capacity, reducing moisture content, and homogenizing feedstock to minimize biomass recalcitrance. Consequently, physical pretreatments induce alterations in specific surface area, crystallinity index, and the degree of polymerization in biomass. The most commonly used techniques include mechanical, microwave-assisted, and ultrasonic pretreatments [61].

Mechanical pretreatment methods encompass milling, grinding, chipping, crushing, and extrusion. *Milling* is primarily utilized for size reduction of the feedstock to achieve particle sizes within the range of 0.2–2 mm. This process influences the physicochemical properties of the biomass by subjecting the raw feedstock to intense mechanical stress, thereby enhancing its reactivity [62]. *Chipping or shredding* is employed when the biomass displays relatively higher resistance and possesses elevated moisture content, resulting in particle sizes ranging from 10 to 33 mm. These techniques can also serve as a preliminary stage before further size reduction. Milling, grinding, and other mechanical pretreatments are primarily aimed at modifying the biomass structure, ultimately reducing biomass recalcitrance. Consequently, the structural features of the biomass are modified, rendering them amenable to subsequent processing steps [63,64]. A study by Yang et al. [65] investigated the impact of size reduction on sugar recovery efficiency from Norway spruce for butanol production. The results indicated that sugar recovery following enzymatic hydrolysis of chipped material was higher than that of milled material. In another study, DeMartini et al. [66] explored how chip size affects the efficiency of steam pretreatment. The findings revealed that larger wood chips are well-suited for rapid decompression methods, such as steam pretreatment, which occurs at lower temperatures and alters accessibility. The advantages of mechanical pretreatments include reduced particle size, increased specific surface area, and improved bulk density. While altering the biomass structure through a single physical process is often insufficient, physical processes are frequently employed either before or in conjunction with chemical and biological processes to attain particle sizes suitable for efficient treatment [67].

*Microwave* irradiation represents a physical pretreatment involving non-ionizing electromagnetic radiation with a frequency falling between infrared and radio waves, employing wavelengths of 0.01–1 m and 300–300,000 MHz. Microwave radiation possesses sufficient energy to induce molecular vibration but lacks the energy required to break chemical bonds. In the presence of a microwave-absorbing material, this energy is irreversibly absorbed, resulting in rapid volumetric heating. This unique heating mechanism confers advantages to the microwave system, including reduced heating time, uniform and selective volumetric heating, and enhanced energy transfer efficiency. Noteworthy benefits of microwave heating over conventional heating methods include lower energy consumption, shorter reaction times, and the avoidance of direct contact with the raw material [63,67]. However, microwave irradiation does come with certain drawbacks, such as high capital investment and elevated energy costs, which must be considered for industrial applications and scale-up strategies [58]. In their study, Muley et al. [68] examined the impact of microwave-assisted delignification and depolymerization of lignin, varying temperatures, residence times, and deep eutectic solvents. Their results demonstrated that microwave heating significantly reduced the required residence time and promoted selective bond cleavage during lignin depolymerization. Additionally, this method led to a narrower distribution of molecular weights compared to conventional heating methods. Another study by Monteil-Rivera et al. [69] compared conventional heating and microwave-assisted lignin extraction, evaluating different techniques under varying conditions. Their findings indicated that under comparable conditions, microwave irradiation resulted in higher lignin yields, such as a 91% yield when using ethanol, and yielded lignin with smaller molecular weights.

### 2.2. Chemical Pretreatments

#### 2.2.1. Alkaline and Acid

*Alkaline pretreatment* involves several essential reactions, including the dissolution of lignin and hemicellulose and the de-esterification of intermolecular bonds. Common reagents such as sodium hydroxide (NaOH), sodium carbonate (Na_2_CO_3_), potassium hydroxide (KOH), and ammonium hydroxide (NH_4_OH) are typically used in alkaline pretreatments, which are conducted under mild conditions. The effectiveness of the process is significantly influenced by factors such as the solid-liquid ratio, temperature, pressure, and residence time. Moreover, this method can be performed at ambient temperature and atmospheric pressure. The primary goal of alkaline pretreatment of lignocellulosic biomass is to break the ester and glycosidic bonds between LCCs and cellulose, as well as the acetyl group bonds of hemicellulose [70]. Disrupting these bonds between LCCs accelerates the modification of the recalcitrant cellulosic structure and facilitates lignin removal. After pretreatment, the resulting solid fraction, often referred to as cellulose-rich pulp, is obtained, while the filtrate contains mainly hemicellulose sugars, lignin, and other inorganic chemicals. In specific alkaline pretreatment approaches, it is possible to recover and reuse chemical reagents. Nevertheless, this method faces challenges related to its long reaction time and the need to neutralize the post-treatment fraction [71,72].

Strong alkaline fractionation has been widely employed for over a century, particularly in the pulp and paper industry. However, a recent trend has been towards adopting mild alkaline fractionation within cellulosic ethanol biorefineries [73]. The degree of solubilization achieved depends on the severity of the operating conditions and the specific lignocellulosic biomass under consideration. Nevertheless, under mild alkaline conditions, solubilization yields ranging from 60% to 80% for both lignin and hemicelluloses can be reasonably achieved [74]. For instance, Geng and Henderson [40] emphasized that alkaline extraction can be readily scaled up and can solubilize 75% of the lignin and 37% of the hemicellulose from corn stover by combining alkaline extraction with ionic liquid (IL) dissolution. In their study, the total carbohydrate content increased from 63% to 87% using this combined process, demonstrating efficient biomass-to-sugar conversion. Moreover, this approach reduces enzyme loading by removing lignin and other extractives prior to IL pretreatment, thus significantly decreasing the enzymatic load required for hydrolysis and maximizing the conversion of biomass polysaccharides into sugars.

*Acid pretreatment* is one of the most widely used techniques for modifying the lignocellulosic matrix by cleaving glycosidic bonds, resulting in the transformation of polysaccharides into oligomeric and monomeric sugars. This technique primarily targets the hemicellulose fraction while also removing the acid-soluble lignin fraction. Additionally, it depolymerizes cellulose into cello-oligosaccharides. However, acid pretreatment has certain drawbacks, such as the degradation of sugars and the decomposition of lignin during the process, leading to the production of various inhibitory compounds for microorganisms, thereby reducing biological and enzymatic activities, including aldehydes, ketones, and phenolic acids, which may not be the desired products [75]. Moe et al. [76] investigated the use of low-value softwood residues for biorefinery applications using a two-step concentrated acid hydrolysis treatment. They emphasized that this approach enables the utilization of low-value softwood residues due to the maturity and relative simplicity of the concentrated acid hydrolysis process. Concentrated acid decrystallization and hydrolysis of lignocelluloses offer a potential alternative for saccharification of xylan-rich lignocellulosic biomass, such as hardwoods. However, it is essential to anticipate some furfural production, which might necessitate detoxification treatment before fermentation if a biological step is included in the process.

During acid pretreatment, organic acids like formic acid or acetic acid, as well as inorganic acids such as nitric acid and sulfuric acid, are used. Acid pretreatment can be applied through various methods. One such method is concentrated acid hydrolysis, which involves using acid solutions with concentrations exceeding 30% to hydrolyze lignocellulose into monosaccharides. This process typically occurs at temperatures below 100 °C, with reaction times ranging from 2 to 10 h under atmospheric pressure. Another method is dilute acid pretreatment, which involves the hydrolysis of hemicellulose into monosaccharides, rendering cellulose more accessible, using acid concentrations around 10% as a catalyst. The reaction conditions for this method include temperatures ranging from 100 to 240 °C, pressures exceeding 10 atm, and reaction times ranging from a few seconds to several minutes. These two methods can be selected for various further valorization steps, such as enzymatic saccharification, fermentation, organosolv, or steam pretreatment [54]. However, concentrated acids are generally less preferred due to their corrosive nature, and their recovery is essential to ensuring the economic feasibility of the pretreatment process in various aspects. For instance, Saha et al. [77] investigated dilute acid pretreatment under multiple conditions and the enzymatic saccharification of wheat straw cellulose and hemicellulose for ethanol and fermentable sugars, respectively. They achieved a 74% saccharification yield with a combined 0.75% (*v*/*v*) H_2_SO_4_ acid pretreatment and enzymatic saccharification without the production of degradation products such as furfural and hydroxymethyl furfural (HMF). They also noted that the formation of sugar degradation products largely depends on the pretreatment temperature. In contrast to concentrated acid treatment, dilute acid treatment necessitates the use of enzymes for saccharification.

#### 2.2.2. Organic Solvent/Organosolv

Organic solvent pretreatment, often referred to as organosolv, employs organic solvents such as ethanol [78,79], acetone [80], glycerol [81], gamma-valerolactone [82], or ethylene glycol [83], to fractionate lignocellulose into high-purity cellulose, lignin, and hemicellulose components. This method allows the separation of cellulose with minimal degradation, resulting in the recovery of cellulose as a solid. In contrast, most of the lignin, water-soluble sugars (mainly hemicellulose-based), sugar degradation products, and other components dissolve into the organic solvent. After pretreatment, the recovery and reuse of the organic solvent are feasible. Organosolv lignin, characterized by its high quality, sulfur-free nature, low molecular weight, preservation of its original composition, and significant amounts of phenolic and aliphatic hydroxyl groups, finds suitability in high-value applications. As a result, the removal of lignin and hemicellulose reduces recalcitrance and increases the surface area of cellulose, thus enhancing enzymatic accessibility for hydrolysis and ultimately improving bioethanol yield during fermentation [39]. In a study by Hage et al. [84], an acid-catalyzed (1.2% H_2_SO_4_, *w*/*w*) ethanol organosolv pretreatment at 190 °C for 60 min was conducted to extract lignin from *Miscanthus*. Comprehensive structural analysis of the extracted lignins, employing techniques such as Fourier-transform infrared spectroscopy (FT-IR), ^13^C and ^31^P Nuclear Magnetic Resonance (NMR), Scanning Electron Microscope (SEM), and Ultraviolet (UV)-spectroscopy, revealed that the primary mechanisms responsible for lignin breakdown were the cleavage of β-O-4 linkages and ester bonds. However, based on the ^13^C NMR and FT-IR spectra, the pretreatment did not substantially impact the structure of lignin.

Organic solvent pretreatment involves using a variety of organic solvents, with or without catalysts, at temperatures ranging from moderate to 240 °C for different residence times (30–240 min) [78,85,86]. Solvents like ethanol and methanol, which have lower boiling points, are favored due to their cost-effectiveness and ease of recovery. Hrůzova et al. [78] used hot water extraction followed by ethanol organosolv to fractionate spruce bark and enhance enzymatic hydrolysis. Their results demonstrated that this combined approach increased cellulose content to 49.6% w/w while reducing lignin content to 25.5%. After enzymatic hydrolysis of the pretreated biomass, the final hydrolysis yield reached up to 70.1%.

Conversely, high-boiling-point alcohols such as ethylene glycol and glycerol require less demanding temperature and pressure conditions, but this comes at the expense of higher energy consumption for solvent recovery compared to lower-boiling-point organic solvents. Another option is the utilization of organic acids, such as formic acid and acetic acid, for biomass treatment under atmospheric pressure. However, acids, while they can serve as catalysts, are known to have corrosive effects in comparison to other organic solvents [87]. The use of acid-catalyzed organic-solvent pretreatment significantly affects the cleavage of internal ether bonds in lignin and the intermolecular bonds between lignin-carbohydrate complexes. The presence of a catalyst can either reduce the required operating temperature while maintaining lignin yield or, for a given temperature, enhance the delignification process [88]. In a study by Imman et al. [89], acid-catalyzed organosolv was investigated for lignocellulose fractionation. They found that adding acid reduced the glucan yield in the solid phase while improving structural homogeneity. The presence of acid catalysts facilitated the hydrolysis of polysaccharide fractions, resulting in increased pentose yield in the aqueous phase and lignin yield compared to non-catalyzed reactions. The use of homogeneous acid promoters enhanced the selectivity of lignocellulose components by breaking glycosidic bonds in the aqueous medium. They observed that different types of acids, such as H_2_SO_4_, HCl, and H_3_PO_4_, at various concentrations, exhibited a similar trend.

#### 2.2.3. Ionic Liquids

Ionic liquids (ILs) are a type of salt composed of an inorganic anion and an organic cation. The cations in ILs consist of organic cores, including ammonium, pyridinium, phosphonium, sulfonium, and cholinium, while the anions can be both organic and inorganic. A noteworthy advantage of ILs is their ability to combine various cations and anions, allowing for the regulation of properties such as hydrophobicity, polarity, and solvent power. Additionally, ILs possess appealing characteristics, including low vapor pressure, high thermal stability, a wide electrochemical range, and remarkable ionic conductivity, even in the absence of water [59,90].

Most ILs remain in the liquid state at room temperature, qualifying them as novel solvents. These room-temperature ionic liquids (RTILs) exhibit a dissolving effect on the lignocellulose matrix, promoting the fractionation of its components and enhancing the production of sugar monomers. This makes RTILs an attractive option for lignocellulose pretreatment and fractionation [91,92]. However, Ghorbani and Simone [93] pointed out that one of the significant challenges of RTILs is their high production costs, which limit their large-scale application. Nevertheless, their study also identified cost-effective starting materials for synthesizing RTILs with desirable characteristics. As mentioned previously, fractionation is a crucial step for efficiently utilizing lignocellulosic biomass, allowing for its conversion into chemicals, dissolution of biomass components, and hydrolysis processes. The use of ILs as dissolution media has garnered attention due to their eco-friendly nature and their ability to enable biomass pretreatment at atmospheric pressure, even at temperatures higher than the boiling point of water.

Recent studies have emphasized the delignification effect of ILs, rendering them suitable as fractionation solvents for lignocellulosic biomass. Various strategies can be employed during or after IL-assisted fractionation to produce fuels, chemicals, and materials. However, it is important to note that ILs are often costly to produce and exhibit high viscosity, resulting in energy-intensive pumping and mixing processes for large-scale applications [94,95]. Fu et al. [42] conducted an evaluation of the solubility of cellulose, xylan, lignin, lignin extractability, and cellulose digestibility in extraction residues using six different ILs. The ILs utilized included 1-butyl-3-methylimidazolium chloride ([bmim]Cl), 1-ethyl-3-methylimidazolium acetate ([emim]Ac), N, N-dimethylethanolamine formate (DMEAF), N, N-dimethylethanolammonium acetate (DMEAA), N, N-dimethylethanolammonium glycolate (DMEAG), and N, N-dimethylethanolammonium succinate (DMEAS). The experiments with ILs were carried out within temperature ranges of 70–150 °C and residence times of 0.5–24 h. Their findings revealed that higher extraction temperatures and longer residence times were more effective for lignin extraction and cellulose hydrolysis of the residues. Notably, the use of [emim]Ac enabled the extraction of 52.7% of the acid-soluble lignin portion from straw at 150 °C for 90 min, resulting in over 95% cellulose digestibility of the residue. [emim]Ac pretreatment achieved a significant delignification rate compared to [bmim]Cl, while DMEAF, DMEAA, DMEAG, and DMEAS proved less suitable. This study suggested the need to optimize extraction conditions and develop an effective IL recycling process to enhance lignin extraction capacity.

#### 2.2.4. Deep-Eutectic Solvents

Deep-eutectic solvents (DESs) have gained recognition as environmentally friendly and cost-effective green solvents for fractionating lignocellulose. The term “deep-eutectic solvent” originates from the significant difference in freezing points observed when two chemicals involved in DES formation are combined. The freezing point of the eutectic mixture is considerably lower than the melting points of the pure components comprising the individual elements. DESs exhibit physicochemical properties similar to those of ionic liquids while being more eco-friendly, affordable, and less toxic than ionic liquids [59,71]. Understanding how the physicochemical properties of DES and the conditions of pretreatment reactions impact the process of fractionating lignocellulosic biomass is crucial for advancing biomass conversion methods. DESs are prepared by mixing two or three components with desired molar ratios of hydrogen bond acceptors (HBAs), such as quaternary ammonium salts, and hydrogen bond donors (HBDs), such as amides and carboxylic acids. The effectiveness of DES pretreatment depends on the synergistic interactions among various process parameters. The characteristics of the raw material and the process variables significantly influence the overall efficiency of pretreatment and the yield of reducing sugar during subsequent enzymatic hydrolysis. Enhancing the efficiency of DES pretreatment involves optimizing key process variables like temperature, time, and the liquid-to-solid ratio [96].

Sun et al. [97] investigated the acidic DES-assisted ball milling pretreatment and its effect on the fractionation of four representative lignocellulose sources with varying cellulose, hemicellulose, and lignin compositions. They employed p-toluenesulfonic acid (p-TsOH) as an HBD and choline chloride (ChCl) as an HBA, using a molar ratio of 2:1, under continuous stirring at 60 °C for 30 min. Subsequently, they mixed 10 g of lignocellulose with 100 g of DES and treated it with planetary ball milling. Afterward, the filtrate was left in a fume hood overnight to precipitate lignin. The study aimed to observe the combined effect of ball milling and DES treatment on lignin extraction. The results indicated that this combined method had a distinct effect compared to traditional chemical methods. The reason for this difference was attributed to the ability of the combined method to disrupt the lignocellulose matrix, resulting in increased cellulose accessibility while preserving the β-O-4 linkages and extracting lignin at near-room temperature. This simultaneous effect was described as a dual function of biomass swelling and lignin dissolution. Furthermore, the cellulose-rich pulp fraction varied for each biomass but displayed enhanced hydrolysis potential. Additionally, the extracted lignins exhibited a substantial number of β-O-4 linkages, which is advantageous for subsequent upgrading processes.

Soares et al. [98] investigated the delignification effects of various aqueous solutions (with 50 wt.% water content) of deep eutectic solvents, such as propionic acid:urea (2:1), urea:choline chloride (2:1), lactic acid:choline chloride (10:1), and p-toluenesulfonic acid:choline chloride (1:1). These solvents were categorized as hydrogen bond donors (lactic acid, propionic acid, p-toluenesulfonic acid, and urea) and hydrogen bond acceptors (choline chloride and urea). Different acid catalysts, like H_2_SO_4_, HCl, and PTSA, were added to facilitate the disruption of the lignocellulose matrix, thereby aiding in delignification and lignin extraction. They applied a mild wood delignification process (90 °C for 8 h) using DES, resulting in the production of a cellulose-rich pulp with a yield of 59.50 ± 0.51 wt.% of the initial wood mass and a Klason lignin content of only 3.86 ± 0.10 wt.%. The study emphasized the importance of using an organic or mineral acid when delignifying under mild conditions, especially when employing lactic acid (choline chloride) and propionic acid (urea) as delignification solvents. In conclusion, an impressive 80.64 wt.% of the initial lignin content in *Eucalyptus globulus* wood could be extracted, and 40.73 wt.% of sulfur-free lignin could be recovered from the DES liquor with this optimized method.

### 2.3. Physicochemical Pretreatments

#### 2.3.1. Steam Pretreatment

The steam pretreatment technique is widely employed as a physicochemical pretreatment method. This method typically operates within a temperature range of 160–240 °C for 1–20 min, subjecting the biomass to high pressure. Key factors such as temperature, residence time, pressure, and the presence of a catalyst significantly influence the steam pretreatment process, directly affecting the final product’s properties. These parameters should be adjusted according to specific pre-treatment objectives. The steam pretreatment process consists of two distinct stages: the autohydrolysis phase and the explosion phase. In the initial stage, the fibril structure, composed of lignin-carbohydrate complexes (LCCs), undergoes significant disruption. Subsequently, thermal energy is converted into mechanical energy [59,99].

The primary effect of steam pretreatment is the opening of the lignocellulose matrix and the modification of LCCs, which promotes the removal of hemicelluloses and improves cellulose accessibility. Therefore, steam pretreatment is primarily used as an initial step in the pretreatment technique for biomass conversion, aiming to enhance enzymatic hydrolysis due to the recalcitrant nature of lignin [100]. Kumar et al. [101] evaluated the enzymatic hydrolysis of steam-pretreated softwood, comprising six different steam-pretreated Douglas-fir wood samples and one sample representing Lodgepole pine. The wood chips were impregnated with SO_2_ (4% *w*/*w* of the substrate) for 12 h, followed by steam pretreatment at 200 °C for 5 min. The steam-pretreated solid fractions were then subjected to enzymatic hydrolysis using a 2% (*w*/*wt*.) acetate buffer at 50 °C and 150 rpm. The experiments involved relatively low (5 FPU/g cellulose) and high (20 FPU/g cellulose) enzyme loadings to assess the effectiveness of steam pretreatment in enzymatic hydrolysis. The study demonstrated that applying the same conditions of steam pretreatment to several different samples resulted in similar chemical compositions, sugar recoveries, and hydrolysis yields. The authors also noted that when the enzyme loading was reduced from 20 FPU/g cellulose to 5 FPU/g cellulose, the hydrolysis yield decreased from 60% to 27%, respectively. These findings indicated that steam pretreatment can enhance the recovery of hemicellulose sugars, primarily hexoses, and improve the ease of hydrolysis of water-soluble fractions. Additionally, the authors suggested that low enzyme loadings would necessitate an additional delignification step to achieve complete hydrolysis. It is also common to use a physicochemical pretreatment that increases the reactivity of the lignocellulosic matrix in fractionation processes. Furthermore, Hongzhang and Liying [102] investigated wheat straw fractionation using combined steam pretreatment and ethanol extraction. GC and HPLC analysis results revealed the presence of organic acids in the hemicellulose sugars, with an overall hemicellulose recovery rate of 80%. The lignin precipitation yield was 75%, and the purity of lignin was determined using infrared spectrometry, reaching a purity rate of 85.3%. Additionally, 85% of the ethanol solvent was successfully recovered. Following the steam pretreatment and ethanol extraction processes, the cellulose recovery rate reached 94%. Electron spectroscopy for chemical analysis and infrared spectrometry showed reduced hemicellulose and lignin content. Their results demonstrated that the combined process enabled the fractionation of each lignocellulose component. Furthermore, the yield and purity of isolated fractions depended on the pretreatment’s severity.

#### 2.3.2. Liquid Hot Water

Liquid hot water (LHW), also known as hot-water extraction, hydrothermolysis, or autohydrolysis, has been utilized as an extraction method for removing hemicellulose from wood to produce pure cellulosic pulp in the pulp and paper industry. Pretreatment involves subjecting biomass to hot water under specific pressure and temperatures exceeding the boiling point, typically within the range of 140–240 °C for 0–20 min [55]. Ruiz et al. [103] comprehensively reviewed the fundamentals of hydrothermal processing, including both steam pretreatment and liquid hot water. They explored the effects and applications of the hydrothermal severity factor in lignocellulosic biomass fractionation and investigated the kinetics and modeling of hemicelluloses in hydrothermal processes. The severity factor (SF) serves as an efficient and applicable parameter that correlates the influence of temperature and time in hydrothermal pretreatment and fractionation of lignocellulosic biomass. Beyond offering insight into industrial applications, the SF is an essential tool for designing experiments and strategies for scaling up. It quantifies how pretreatment conditions, specifically temperature and time, affect the lignin removal and the recovery of sugars from various feedstocks, thereby explaining the process efficiency. Higher severity in pretreatments involves elevated temperatures and longer durations, resulting in increased lignin exposure and degradation, albeit with the generation of inhibitory compounds. Additionally, the adjustment of pretreatment intensity leads to the removal of amorphous components like hemicellulose and lignin, impacting the presence of lignin and phenolic compounds.

LHW pretreatment is akin to steam pretreatment, except it utilizes water in a liquid state at desired temperatures instead of steam. Wojtasz-Mucha et al. [104] examined the effects of LHW and steam pretreatment on Norway spruce at various residence times. Steam pretreatment was carried out at 150 °C for 15 and 30 min, with a water-to-wood ratio of 4:1. In contrast, LHW experiments were conducted at the same temperature and water-to-wood ratio as steam pretreatment for 15, 30, 60, and 90 min. The results revealed that the explosion step in steam pretreatment significantly influences the sample’s composition, promoting mass transport and achieving hemicellulose removal comparable to LHW. On the other hand, shorter residence times in LHW have a significant impact on the local composition of the treated wood.

LHW pretreatment uses the advantages of the acidic properties of water, facilitated by the dissociation of its hydronium ions, promoting the hydrolysis of lignocellulose at the desired temperatures. This results in the cleavage of internal chemical bonds like glycosidic and aryl-ether bonds in biomass due to changes in hydronium ion concentration and modulated hydrogen bonding under high temperature and pressure. LHW pretreatment induces hemicellulose hydrolysis and lignin removal, enhancing cellulose accessibility while minimizing the formation of fermentation inhibitors typical at higher temperatures. LHW pretreatment yields higher hemicellulose sugar and cellulose digestibility, a critical factor for scaling up applications and reducing pretreatment costs. Moreover, this technology enhances safety and environmental considerations due to reduced chemical usage [56,105]. Wojtasz-Mucha et al. [106] conducted birch treatments at 130, 150, and 170 °C for 30, 60, and 120 min. At 130 °C, the extraction yield of total dissolved solids increased from 2.6% to 10.8%, with an increase in residence time from 30 to 120 min. Results showed that extraction yields increased more significantly with increasing temperature and residence time at 150 °C and 170 °C. For the 120 min, the extraction yield increased to 33.7% and 43.8% at 150 °C and 170 °C, respectively. This increase in extraction yield can be attributed to acidic autohydrolysis and component dissolution at higher temperatures. The study also suggested a possible link between lignin depolymerization, hemicellulose extraction yield, pH, and temperature, driven by deacetylation and ongoing hydrolysis reactions, leading to the formation of hemicellulose with varying molecular weight ranges, lignin in the liquid phase, and sugar degradation products.

#### 2.3.3. Ammonia Fiber Explosion

The Ammonia Fiber Explosion (AFEX) technique shares similarities with the steam pretreatment method, but instead of using steam, it involves treating biomass with liquid ammonia at moderate temperatures, typically ranging from 60 to 100 °C, for residence times between 30 and 60 min, all under high pressure (approximately 1.72–2.06 MPa). The combination of elevated pressure and temperature triggers rapid ammonia expansion, leading to biomass swelling and physical disintegration. Furthermore, this process partially disrupts the crystalline structure of cellulose and lignin. AFEX is effective in modifying or reducing the lignin fraction in lignocellulosic biomass while leaving the hemicellulose and cellulose components relatively unaffected [99,107]. AFEX boasts several advantages, including the absence of inhibitory by-product formation, efficient sugar recovery, no need for additional size reduction steps or post-processing water washing, and the use of liquid ammonia as a nitrogen source for subsequent microbial fermentation. It is worth noting that ammonia recycling after pretreatment is essential to reducing costs and minimizing the environmental impact. However, AFEX does have certain limitations when compared to alternative processes. It is particularly effective on biomass with lower lignin content and does not significantly solubilize hemicellulose, unlike methods such as dilute-acid pretreatment [108].

Zhang et al. [109] conducted an investigation into the combined AFEX and NaOH (A-NaOH) pretreatment on various types of lignocellulosic biomass, including fountain grasses, oak, and camphor wood, representing herbaceous, hardwood, and softwood sources. The impact of the pretreatment on these materials was assessed through an enzymatic efficiency analysis. In the AFEX step, biomass was subjected to conditions of 130 °C with a liquid ammonia-to-biomass ratio of 1:1 for 10 min, followed by rapid pressure release. Subsequently, the materials were treated with various NaOH solution concentrations at 80 °C for 40 min. In this NaOH step, the solid-to-liquid ratio was 1:10, and NaOH concentrations of 1%, 2%, 3%, 4%, and 5% *w*/*v* were employed. Notably, under the 4% A-NaOH conditions, the results demonstrated a substantial increase in lignin removal (84.2%, 59.7%, and 36.7% for *Pennisetum sinese*, oak, and camphor wood, respectively) and enhanced enzymatic efficiency (36.2%, 9.7%, and 6.5% for the same wood types). Furthermore, analysis using FT-IR and NMR indicated that the combined pretreatment method effectively disrupted the lignin-hemicellulose bonds, resulting in efficient lignin removal. XRD and SEM analyses also illustrated that the pretreatment efficiently removed amorphous components, promoting subsequent enzymatic hydrolysis and enhancing cellulase accessibility.

### 2.4. Biological Pretreatments

Biological pretreatment is an approach that uses microorganisms to modify lignocellulosic biomass, enhancing accessibility to cellulose, hemicelluloses, and lignin for the production of diverse products. This method is characterized by its high selectivity, minimal chemical usage, and low energy requirements compared to other techniques. It is also environmentally friendly, taking place under mild conditions with no adverse effects from the generated by-products [110]. Tian and Zhou [111] conducted a comprehensive review of various biological pretreatment processes, including anaerobic digestion, enzymatic pretreatment, and microbial pretreatment, while also delving into different sub-categories within biological pretreatment. Key parameters influencing the successful biological conversion of lignocellulosic materials include cellulose crystallinity, accessible surface area, protection of lignin and hemicellulose, cellulose degree of polymerization (DP), acetylation degree of hemicelluloses, cellulase adsorption and desorption, and biomass swelling capacity. In recent years, interest in harnessing the potential of biological pretreatments for the production of biofuels and value-added products from biomass has significantly increased [112,113,114]. Sindhu et al. [115] explored the involvement of microorganisms in the degradation of polysaccharides during biological pretreatment. Their paper provided a comprehensive examination of various process parameters that influence the efficiency of the process and offered valuable insights into future prospects in this field. The authors underscored that while the advantages of biological pretreatment over traditional chemical methods are evident, addressing drawbacks such as the relatively slow process and partial hemicellulose hydrolysis is crucial when considering commercial application. They emphasized the need for extensive research and development, as well as the importance of reducing the cost of pretreatment and enzymatic saccharification systems. Additionally, the design of reactors that minimize heat generation during biological pretreatment and the use of advanced molecular techniques to identify efficient lignin-hydrolyzing microbes are essential aspects to be addressed in this field.

## 3. Two-Step Fractionation for Lignin Extraction and Its Valorization

Delignification is a pivotal step in the fractionation process, exerting a direct impact on lignin purity and its properties. Developing an effective and distinctive strategy for lignin isolation or extraction with high purity is of paramount importance. Single-step delignification pretreatments, such as organosolv [48,84,116,117], ionic liquid [90,118,119,120], and alkaline or acid-catalyzed pretreatments [40,81,121,122,123], are widely recognized for their efficacy in lignin extraction. Nevertheless, these single-step pretreatments come with a range of technical and environmental challenges due to their prolonged reaction times and elevated energy consumption. These factors limit their applicability for large-scale processes and the potential benefits of biofuel production. Additionally, when single-step methods are employed, the primary focus tends to be primarily on cellulose, often neglecting the generation of significant volumes or co-product values during manufacturing. This is due to single-step methods falling into two categories: either cellulose is substantially purified by removing other components, or some or all components coexist with cellulose until the final product is obtained. In cases where various components are not significantly recovered, claiming their recovery is inaccurate. Furthermore, the purification process to obtain pure cellulose often involves converting, contaminating, or degrading the other lignocellulose components into low-value materials, necessitating costly sub-operations such as energy recovery or wastewater treatment [124].

Presently, the methods employed to isolate lignin from the lignocellulosic matrix can be broadly categorized into two groups based on the type of lignin produced: sulfur lignins (such as Kraft, sulfite, and enzymatic hydrolysis processes) and sulfur-free lignins (including organosolv and soda pulping processes). Combining these pretreatment methods with other techniques offers a viable approach to enhancing the fractionation of each lignocellulosic component. This approach allows for the creation of separate process streams, each tailored for extracting specific compounds, whether they be lignin, hemicellulose, desirable sugar streams, or cellulose. However, combining different pretreatments should be approached cautiously, as implementing multiple pretreatments with varying applications and operating conditions can lead to additional costs and practical issues. Various pretreatment methods, such as physical-chemical, physical-biological, physical-alkaline, microwave-chemical processing, and physical-physicochemical-chemical treatment, can be combined to enhance fractionation, especially when selective fractionation is applied [54,59,125]. Consequently, the relatively labile nature of hemicellulose encourages the use of a two-step pretreatment approach, where hemicellulose is first solubilized and recovered under mild conditions before lignin extraction. The extraction of high-purity lignin from lignocellulosic biomass becomes essential when considering subsequent utilization processes, which can serve as a platform for value-added products, chemicals, and fuel or fuel additive industries to efficiently leverage these technologies. Nonetheless, extracting all lignin in its native state, particularly when considering the lignin-carbohydrate complexes, remains challenging [126].

The efficiency of lignin extraction and its properties, such as final structure and aromatic content, can be influenced by several factors, including the type of biomass, extraction methods, and process severity. These factors, in turn, have a profound impact on the potential valorization pathways for lignin into value-added products. It is important to note that lignin structure and characteristics can vary significantly among different sources, presenting a challenge in obtaining lignin in its native form. However, this variability also underscores the complexity of lignin extraction methods and their subsequent industrial applications [127]. Lignin research typically falls into two distinct sub-categories. The first involves the analysis of lignin, its structural relevance to other substituents, and the extraction methods employed. The second sub-category focuses on the applications of lignin in various fields. Research and review papers have been published exploring the utilization of lignin for value-added chemicals [29,128,129], biopolymers [130,131,132,133], and lignin-based carbon applications [134,135,136]. Additionally, several reviews have concentrated on lignin conversion technologies, addressing different pathways and strategies for fuel production [137,138,139,140,141]. For instance, Sures et al. [142] reviewed lignin transformation into various forms of fuel and particularly emphasized the availability of lignin waste in the Indian subcontinent. They highlighted the feasibility of generating solid, liquid, and gaseous fuels from lignin through different pretreatment strategies, with a focus on the potential for liquid fuel production. In another study, Luo et al. [143] discussed the conversion of lignocellulosic biomass into fuels and chemicals, presenting a lignin-first biorefinery strategy. Their primary focus was on exploring downstream processes for lignin degradation products and carbohydrate residues, primarily for the production of liquid fuels. Understanding the chemical composition of lignin, the effects of pretreatment on its chemical structure, and its characteristics are crucial for gaining insights into lignin extraction and its potential applications.

In the quest for improved and selective fractionation of lignocellulose, *with a primary focus on lignin extraction/isolation*, two-step pretreatment methods have been explored. Examples of these approaches include organosolv/steam pretreatment, alkaline/deep eutectic, or alkaline/dilute acid. A summary of different combined pretreatment methods for lignin extraction and their delignification yields can be found in Table 4.

In a study by Das et al. [144], the structural effects of one- and two-step pretreatments, followed by either dilute acid or acid-catalyzed steam pretreatment, on isolated lignin were compared. The authors noted that alkaline pretreatment could modify the lignin structure, leading to an increase in high-molecular-weight constituents, as evident in their gel permeation chromatography (GPC) results. The heteronuclear single quantum coherence spectroscopy (HSQC) NMR spectra of treated samples revealed that acidic conditions could break β-O-4 bonds, predominantly causing the formation of G-units of monolignols and bond breakage. Furthermore, ^31^P NMR spectra indicated that pretreatment severity could determine the alteration of the lignin structure through the depolymerization and repolymerization of its subunits. The authors emphasized that a comprehensive understanding of the structural changes in lignin brought about by the applied pretreatments is crucial for future applications and valorization. In another study, Watkins et al. [145] explored lignin extraction from various lignocellulosic biomasses using a sequential organosolv method. The aim was to assess the potential usage of phenol precursors in resole phenolic systems as a partial replacement. Lignin was extracted from wheat straw, pine straw, alfalfa, kenaf, and flax fiber using two distinct pretreatment strategies: formic acid/acetic acid (FA/AA) pretreatment or peroxy-formic acid/peroxy-acetic acid (PFA/PAA) pretreatment. Following the extraction step, the isolated lignins were purified for characterization. FT-IR spectra exhibited uniformity across all extracted lignin samples via organosolv, reflecting their chemical structure. When comparing the yield of lignin extraction, alfalfa produced the highest at 34%. DSC and TGA analyses were employed to compare the thermal behavior of the extracted lignins. DSC analysis revealed a higher heat of reaction for lignin from flax fiber (190.57 J/g) and alfalfa (160.90 J/g). The authors noted that the thermal properties of lignin samples were influenced by their source. TGA was employed to study biomass degradation, with wheat straw-derived lignin exhibiting the highest thermal stability, resulting in a char yield of 40.41%. Following wheat straw, flax fiber (39.22%), alfalfa (35.04%), and pine straw (29.45%) exhibited decreasing thermal degradation, leading to the formation of char on the surface. This enhancement is attributed to the chemical structure of the lignin samples, making them valuable as partial replacements in phenolic resin systems with improved thermal properties.

**Table 4:** A summary of various two-step pretreatment studies.

Pretreatment	Feedstock	Conditions	Remarks of Studies	Lignin-Based Results	References
Two-step/pre-extraction and organosolv	Black spruce (*Picea mariana*)	Prior treatment with ethanol: water mixture in reflux reactor at 80 °C for 6 h; further treatment with EtOH: H_2_O ratios (50:50, 60:40, 70:30, and 80:20) for different temperatures (160, 180, and 200 °C) and residence times (60, 90, and 180 min)	Recovery yield reached 74% with pre-extraction from an average of 70%	62% of the lignin precipitated	[86]
Two-step/Steam pretreatment and enzymatic mild acidolysis lignin extraction	Corncob residue (*Zea mays*)	Soaked with 0.5 wt.% H_2_SO_4_ for 12 h, steam pretreatment was at 180 °C for 10 min with 1.0 MPa, milling for 10 h, and cellulase concentration of 45FPU g^−1^ at 50 °C, 180 rpm for 72 h, and further rotary evaporated (45 °C, 90 bars)	Extracted lignin from CRSE EMAL contained a decent amount of β-O-4′ linkages, and it provides a good base for further lignin utilization	99% purity of lignin, and the yield was 57.3%	[146]
Two-step/Milling and GVL-water fractionation	*Eucalyptus globulus*	Chips were ground to sawdust, and only those smaller than 125 microns were collected and further treated at 180 °C for 120 min	Precipitated lignin had a high phenolic content, relatively low polydispersity, and low molecular mass	50–60% of the extracted lignin was precipitated	[147]
Multi-step/Steam pretreatment, enzymatic hydrolysis, and GVL-fractionation	Cornstalk (*Zea mays* L.)	Material treated with steam pretreatment @1.5 MPa for 5 h, then treated with 30 U/g cellulase loading at 50 °C for 48 h. Lastly, lignin was sequentially fractionated using GVL at water ratios of 60:40 (*v*/*v*), 40:60 (*v*/*v*), and 5:95 (*v*/*v*), respectively	The obtained three lignin fractions’ molecular weight had gradually decreased, and functional group contents increased with this phenomenon	Sequential lignin fractionation resulted in 41.10%, 29.13%, and 24.37%, respectively	[148]
Two-step/Alkaline extraction and black liquor precipitation	Sugarcane bagasse (*Gramineae Saccharum officinarum* L.)	Alkaline extraction was conducted using 6% *w*/*w* NaOH for 1 h at 90 °C with a liquid-solid ratio of 15. The obtained solid fraction was washed until the pH reached neutral, and then the obtained black liquor was precipitated using mineral and organic acids at 45 °C up to a final pH of 4	The precipitation yields of black liquor ranged from 9 to 15%; lactic acid had the highest value	The solubilization and delignification yields were up to 53% and 81%, respectively	[128]
Two-step/Milling and oxy-organosolv	Wheat straw (*Triticum aestivum*)	First, the straw was pretreated by removing the peel and cutting it up. 1 to 5 mm and 100–500 μm sizes of straw were used for further The water-to-ethanol ratio was 3:7 (*v*/*v*) to 1:9 (*v*/*v*), and the straw-to-liquid ratio was 1:15 to 1:25 with a temperature range of 70 °C to 90 °C and a rate of 2 °C/min for 1 h @1000 rpm	Continuous oxygen flow was used to contain the inside pressure @ 0.8 MPa and lignin fractions were precipitated with and without oxygen assistance	The lignin yield was achieved at 46% with oxygen assistance @ 90 °C, and the range of lignin yield was between 19 and 46%	[149]
Two-step/Hydrothermal pretreatment and organosolv	Sweetgum (*Liquidambar styraciflua*)	First, the wood chips were pretreated at 180 °C for 40 min with a liquid/solid ratio of 4. Acetone, methanol, and acetone/methanol mixture (6:1, *v*/*v*) were used for extraction according to the solvent’s boiling point for 8 h	The extracted lignin had a low molecular weight, high phenolic hydroxyls, and low native lignin interunit linkages	Lignin yields ranged from 26.9% to 33.2%	[150]
Two-step/Alkaline and combined alkaline and acid pretreatment	Jerusalem artichoke stalks (*Helianthus tuberosus* L.)	Raw material was first treated with 2% (*w*/*v*) NaOH at 121 °C for 30 min. After, the spent filtrate was concentrated using H_2_SO_4_ (98.3%, *w*/*v*) at 60 °C and then kept at 70 °C for 1 h to precipitate lignin.	In the first step, 57–69% of the lignin was removed, and this study’s main aim was to increase enzyme accessibility. The results showed that the two-step approach was significantly better than the single-step approach	Lignin recovery yield was 36.78%	[151]
Two-step/Mannitol (MT) assisted p-toluenesulfonic acid/pentanol pretreatment	Poplar chip (*Populus*)	TsOH/pentanol with a solid-to-liquid ratio of 1:10 was used at 120 °C for 40 min. Simultaneously, different concentrations of MT were loaded into the pretreatment, and experiments were carried out under the same conditions.	The lignin obtained from the organic phase during pretreatment showcased β-O-4 bond characteristics akin to those found in native cellulosic enzyme lignin.	In the presence of 5% MT, the delignification rate reached 29%.	[152]
Two-step/Alkaline and deep eutectic solvent pretreatment	Bagasse (*Gramineae Saccharum officinarum* L.)	For alkaline pretreatment, 8 wt.% of NaOH was used at 90 °C for 2 h with a solid-to-liquid ratio of 1:30. After the alkaline step, La/ChCl was used as DES solution with alkaline extracted solid-to-DES (1:25) at 110 °C for 12 h.	The results of this study showed that separating lignin after DES recycling and reuse was possible.	The lignin removal rate was 86.7%	[43]
Two-step/Alkaline deep eutectic solvent and sequential acid precipitation	Wheat straw (*Triticum aestivum*)	The mixture of glycerol and K_2_CO_3_ with a molar ratio of 5:1 was used as alkaline DES. The material was transferred to K_2_CO_3_-Gly DES at 3 wt.% and stirred at 100 °C for 16 h. For acid-precipitated fractions, the spent liquor was concentrated to pH 2 using HCl.	Using sequential acid precipitation, three different lignin fractions were extracted from DES lignin, and the purity of each fraction was improved, respectively.	DES lignin was precipitated in a pH-6 condition at 59% wt.	[153]
Two-step/Ionic liquid and acid pretreatment	Kraft lignin	As a raw material, Kraft lignin was pretreated at 80–160 °C for 30–150 min and then concentrated to pH 2–3 using HCl. The solid fraction that was precipitated from spent liquor is called regenerated lignin.	The results showed that the effect of temperature was more essential than the residence time. FT-IR results also showed that there were no major differences between kraft lignin and regenerated lignin.	The lignin degradation rate was up to 27%, and it showed that the strong acidity of ionic liquids could destroy the lignin structure while increasing its degradation rate.	[154]
Two-step/organic solvent and solid organic acid combination	*Hybrid poplar*	Material treated with a mixture of p-TsOH (30 mL) and GVL-H_2_O (95:5, m/m) at different temperatures (60–100 °C) for 30, 45, 60, and 90 min.	The isolated lignin has a low molecular weight with a high phenolic hydroxyl group content	Lignin removal up to 86.14% under optimum conditions	[155]
Two-step/pysical and chemical pretreatment	Poplar chip (*Populus*)	Wood chips were treated with steam in a twin-extruder for 5 min. After, treated wood chips were added to a p-TsOH (60% and 70% wt.) solution and heated at 70 and 80 °C for 1 h	The isolated lignin had a high hydroxyl content, higher β-O-4 aryl ether linkages, and narrow polydispersity	Lignin removal was between 65–85%	[156]
Two-step/Ball milling and GVL-assisted fractionation	Pinewood (*Pinus sylvestris* L.)	The material was subjected to milling for 20 h and then treated with 80% aqueous GVL at different temperatures of 140, 160, and 180 °C for 2 and 4 h	The highest lignin yield was obtained at 180 °C for 4 h with 50% solid recovery	Lignin yield ranged between 3–33%	[82]
Two-step/Hybrid steam pretreatment and organosolv	Spruce (*Picea abies*)	200 g of spruce was mixed with 400 h of ethanol and manually fed into the hybrid reactor. After that, 52% *v*/*v* of ethanol was loaded into the reactor. The reactor heated up to 200 °C for 30 min	This study has comparable results for hardwood using the same hybrid pretreatment method for the production of phenolics and aromatics	Isolated lignin had 65% wt. of C content with a very low sulfur content	[157]
Two-step/combinatorial pretreatment of dilute acid, liquid hot water, sodium hydroxide, and ethanol and sequential fermentation step	Corn stover (*Zea mays* ssp. *mays* L.)	First step: the material was pretreated with dilute sulfuric acid or liquid hot water with a 10% (*w*/*w*) solid loading. Second step: the pretreated solid fraction loaded as in the first step and pretreated with NaOH or/and ethanol at different conditions. The liquid fraction, which was lignin-rich, was collected for lipid fermentation	The results showed that the combinatorial pretreatment, together with fermentation optimization, improved lipid production while using lignin as the carbon source	Alkaline fractioned lignin as a potential carbon source	[158]
Two-step/Alkaline pretreatment and acid precipitation	Bamboo chips (*Bambusa vulgaris)*	Wood chips were treated by different NaOH conditions (0.1–1.0%), various solid loadings (5–15%), and various residence times (60–240 min) at 120 °C. Acid precipitation was carried out to the pretreated material by adjusting the pH to 2 using 2 M HCl	The statistical model showed that the optimum pretreatment conditions were: 1.3% (*w*/*v*) NaOH concentration, 10% (*w*/*v*) solid loading, and 150 min of alkaline pretreatment	Soda lignin recovery 104.6 mg/g of biomass	[159]
Two-step/Aqueous ammonia and dilute acid pretreatment	Rice straw (*Oryza sativa*)	The first step was performed at 100–190 °C and 8 mL/min for 20 min using 15 wt.% aqueous ammonia, and the second step was performed at 130 °C and 8 mL/min for 20 min using sulfuric acid	The first stage was to remove the lignin selectively	The delignification rate for the two-step strategy varied between 69.2% and 83.6%	[160]
Two-step/Liquid hot water and imidazole treatment	Elephant grass (*Pennisetum purpureum*)	LHW pretreatments were performed at 160 °C, 180 °C, 200 °C, and 220 °C under non-isothermal conditions for 60 min. The second step, imidazole treatment, was carried out at 140 °C for 182.5 min	The results showed that the combination of these pretreatments promotes the use of less severe conditions during hydrothermal pretreatment	Resulted in an 83.8% delignification rate	[161]

In a study conducted by Wang et al. [162], a variety of analytical techniques, including NMR, FT-IR, UV, SEM, and TGA, were employed to explore the influence of residence time on lignin fractionation. The study focused on *Lespedeza cyrtobotrya stalks*, a member of the pea family, and employed a two-step fractionation approach. The process began with steam pretreatment of the stalks at 2.25 MPa, with residence times ranging from 2 to 10 min. Subsequently, the treated material was subjected to a 1% NaOH aqueous solution at 50 °C for 3 h. This comprehensive fractionation method allowed the isolation of all major lignocellulosic components, including cellulose, hemicellulose, and lignin. Of particular interest was the examination of the isolated lignin fractions to assess the impact of residence time during the steam pretreatment on the fractionation process and the resulting lignin characteristics, crucial for evaluating its potential in value-added product applications. The findings revealed that steam pretreatment significantly enhanced lignin isolation compared to single-step alkaline pretreatment. Lignin isolation yield increased dramatically, rising from 0.47% to 17.13%, through the adoption of the two-step fractionation process. Notably, the severity of the steam pretreatment was found to influence the lignin yield, with lower severity conditions yielding lignin with a larger surface area, as observed through SEM analysis. However, as the severity of the steam pretreatment increased, several repolymerization reactions involving lignin and sugar degradation products occurred, leading to reduced lignin solubility.

Panagiotopoulos et al. [163] conducted an investigation into the impact of sequential pretreatment methods on hemicellulose and lignin recovery, as well as the enrichment of cellulosic substrates. Their experiments were carried out under mild conditions, involving steam pretreatment and acid-catalyzed organosolv techniques. They emphasized that the use of mild steam pretreatment prevented lignin solubilization and improved the subsequent organosolv delignification process when lower temperatures and reduced acid loadings were employed. The focus here was on maximizing the solubilization and recovery of the hemicellulose component through the initial steam pretreatment phase. Subsequently, they evaluated the ability of the mild organosolv treatment to extract lignin effectively in a reactive form while enhancing the hydrolysability of the cellulosic fraction. The results demonstrated that when hemicellulose was not removed in the initial stage and a one-step ethanol organosolv pretreatment was employed with sulfuric acid as a catalyst, 64% of the original xylose remained in water-soluble and insoluble fractions. Subsequently, when acid-catalyzed organosolv pretreatment was applied at 170 °C for 30 min with the addition of 1% H_2_SO_4_ following the initial steam pretreatment, a significantly high total xylose recovery of 89% was achieved. Moreover, contrary to concerns regarding the interference of steam pretreatment with subsequent organosolv delignification, prior steam treatment actually improved lignin solubilization, with over 66% of the original lignin effectively removed through the two-step pretreatment. Additionally, the analysis of the extracted lignins, employing UV–VIS spectroscopy and NMR techniques, revealed that the number of functional groups in the lignin structure remained comparable to levels observed with single-step organosolv pretreatment. Furthermore, the cellulose-rich fraction obtained after the process, insoluble in water and organosolv solvent, exhibited a high hydrolysis efficiency, achieving nearly 90% conversion with an enzyme loading of 5 FPU per gram of cellulose. These findings underlined the positive impact of employing mild steam treatment to solubilize and recover a substantial portion of hemicellulose in a water-soluble fraction before applying organosolv pretreatment. This approach not only facilitated lignin recovery in a usable form but also enhanced enzyme accessibility to the enriched cellulosic component. However, it is important to note that during the organosolv pretreatment, a significant portion of hemicellulose tends to dissolve into the ethanol-rich cooking liquor, presenting challenges for downstream lignin recovery. Nevertheless, mild steam pretreatment conditions appeared to make the removal and fragmentation of the lignin macromolecule easier during the subsequent organosolv pretreatment.

Another two-step fractionation study, conducted by Gelosia et al. [164], delivered promising results in terms of delignification, supporting the recovery and valorization of all lignocellulosic components. The study compared three extraction techniques that utilized gamma-valerolactone (GVL) as a solvent: Soxhlet extraction, microwave-assisted extraction, and an open vessel on a hotplate stirrer. These techniques were combined with a steam pretreatment step before application. The findings showed that the combination of steam pretreatment and GVL organosolv yielded favorable outcomes for common reed delignification, with delignification rates ranging between 75% and 78%. Moreover, cellulose enrichment was achieved with approximately 100% retention. The crucial role of steam pretreatment in common reed fractionation was evident in the marked enhancement of delignification. The severity of the steam pretreatment significantly transformed the biomass structure, making lignin extraction more efficient, regardless of variations in experimental conditions such as residence time (30–120 min), temperature (90–200 °C), and extraction methods. While all extraction techniques demonstrated favorable outcomes concerning these variables, microwave-assisted extraction (MAE) stood out as the least time-intensive and most effective method for controlling process conditions. The study highlighted the potential of this two-step process for the recovery and valorization of all biomass components, particularly for applications requiring materials with a high cellulose content. In another study, Wufuer et al. [154] explored a two-step strategy involving ionic liquids (ILs) and hydrothermal liquefaction for the conversion of lignin into value-added chemicals. The study investigated the impact of pretreatment conditions on lignin using a selection of ILs, including N,N-dimethyl hydrogensulfate, N,N-dimethylacetate, and N,N-dimethyl hydrochloride. The results underscored the substantial influence of temperature and acidity levels of the ionic liquid on lignin degradation and deoxidation during the pretreatment. At a temperature of 160 °C for 60 min, the lignin degradation yield reached 25.58%, with a deoxidation yield of 37%. The analysis of lignin degradation products revealed the high selectivity of acidic protic ionic liquids in cleaving the β-O-4 bonds within the lignin structure, resulting in a notable yield of vanillin at 59.16%. Additionally, the acidity of the ionic liquids not only influenced the selectivity of degradation products but also played a pivotal role in modifying the structure of regenerated lignin. Adding the ionic liquid step was found to enhance the yield of hydrothermal liquefaction by 27% when compared to single-step hydrothermal liquefaction. Therefore, the pretreatment of lignin with acidic protic bio-based ionic liquids holds promise as an efficient method for lignin degradation and the production of value-added chemicals.

Sathitsuksanoh et al. [165] delved into the characteristics of extracted lignin using a two-step pretreatment method on wheat straw, Miscanthus, and Loblolly pine. All three feedstocks produced precipitated lignin after IL pretreatment, in both solid precipitate and soluble forms in the supernatant. Additionally, lignin was extracted after enzymatic hydrolysis, and all these forms were compared with the solid precipitate. The results revealed that solid precipitates and extracted lignins after enzymatic hydrolysis exhibited high molecular weights at lower severity conditions. This indicated that lignin with varying molecular weights could be separated into distinct process streams. It was observed that ionic-liquid pretreatment predominantly generated precipitated lignin with higher molecular weights, leaving lignin with lower molecular weights in the solution, available for further extraction. NMR results indicated that all three feedstocks underwent varying levels of depolymerization. The number of β-O-4 linkages per 100 aromatic units in the solid precipitate and extracted lignin decreased compared to the initial biomass after enzymatic hydrolysis. However, when treated at 120 °C, the lignin in the solid precipitate and extracted lignin displayed signs of depolymerization for all three feedstocks, with only a slight reduction in β-O-4 linkages. Furthermore, there were no noticeable alterations in lignin interunit linkages in pine following pretreatment at different severities. This indicated that pine might exhibit a higher level of resistance to breakdown compared to the other two feedstocks, primarily due to the more condensed nature of its lignin aromatics.

## 4. Conclusions

Lignin, a well-branched amorphous biopolymer that provides rigidity to plants, represents one of the key components of lignocellulose and serves as a valuable source of aromatic compounds. This review delves into the realm of two-step fractionation for efficient lignin extraction, offering insights into lignin-based valorization pathways aimed at sustainable and environmentally friendly bioproducts found within the existing literature. The necessity for sustainable development and the reduction of reliance on fossil-based industries for chemicals, fuels, fuel additives, and energy have underscored the significance of harnessing the individual components of lignocellulose, each with its own unique potential for diverse product streams. A multitude of pretreatment techniques exist to bolster the efficiency of lignocellulosic biomass utilization, yet it is important to recognize that these methods often align with distinct objectives. Beyond the nature of the lignocellulosic source and its specific composition, an array of parameters must be considered when evaluating the viability of pretreatment and subsequent biorefinery processes, including energy consumption, cost factors, solvent requirements, catalyst expenses, and overall operational costs essential for shaping these processes for practical industrial implementation. Nonetheless, a deeper comprehension of this subject and further research are imperative to gain a more profound insight into the extraction of lignin and its potential applications. One of the primary challenges in this pursuit lies in transitioning from laboratory experiments to large-scale industrial utilization. To expedite this transition, fostering increased collaboration and partnerships between the scientific and industrial communities is of paramount importance.

## Figures and Tables

**Table 1:** Major components of lignocellulose from different sources (dry basis, %) [6,13,14,15].

Biomass	Type	Cellulose	Hemicellulose	Lignin
Spruce	Softwood	44	29	27
Birch *	Hardwood	42	38	19
Wheat straw *	Herbaceous	40	21	20
Aspen *	Hardwood	53	22	20
Oak *	Hardwood	38	29	25
Pine *	Softwood	41	26	27
Hemlock	Softwood	42	32	26
Bagasse *	Herbaceous	39	29	19

* The rest of the biomasses are extractives and ash.

**Table 2:** Content of lignin sub-units for different lignin types [28,29,30].

	Content (%, *w*/*w*)		
Type of Lignin	H-units	G-units	S-units
Herbaceous	5–30	35–80	20–55
Softwood	-	90–95	5–10
Hardwood	0–8	25–50	50–75

**Table 3:** Comparison of pretreatment methods (Adapted from [60]).

Pretreatment Method	Advantages	Drawbacks
Physical	Minimize the structural recalcitrance	Lack of ability to remove lignin
	Reduce particle size and moisture content	Higher energy-demand
	Increase the accessibility and storage availability	Insufficient separation of components
Chemical	Room temperature	Higher cost
	Higher delignification rates	Certain effects on the environment and fermentation
	Maximize conversion of polysaccharides into sugars	
	Fast	
Physicochemical	Improve the accessibility of the lignocellulosic matrix	High demand for energy
	Lack of formation of inhibitors	High cost
	Lignin removal efficiency	Higher temperature and pressure
Biological	Lower energy consumption	Low efficiency
	Lignin and hemicellulose degradation	Low rate of hydrolysis
